# Differential effects of oral and transdermal menopausal hormone therapy on prostacyclin and thromboxane in platelets

**DOI:** 10.1002/phy2.275

**Published:** 2014-03-26

**Authors:** Limor Raz, Larry W. Hunter, Muthuvel Jayachandran, John A. Heit, Virginia M. Miller

**Affiliations:** 1 Department of Surgery, Mayo Clinic, Rochester, Minnesota, 55905; 2 Department of Physiology and Biomedical Engineering, Mayo Clinic, Rochester, 55905, Minnesota; 3 Department of Internal Medicine (Division of Cardiovascular Diseases), Mayo Clinic, Rochester, 55905, Minnesota

**Keywords:** Endothelial function, estrogen, estrone, KEEPS

## Abstract

Menopausal hormone therapies (MHT) may increase thrombotic risk but modulate endothelial function and reduce development of vascular lesions. This study compared effects of MHT on prostanoid‐modulated adenosine triphosphate (ATP) secretion from platelets in relationship with endothelial reactive hyperemia (RH) index and carotid intima medial thickness (CIMT). Participants were healthy, recently menopausal women of the Kronos Early Estrogen Prevention Study (KEEPS) randomized to one of three treatments: oral conjugated equine estrogen (oCEE, 0.45 mg/day), transdermal 17*β*‐estradiol (tE2, 50 *μ*g/day) each with intermittent oral progesterone or placebo pills and patch (PL). Prostacyclin and thromboxane A_2_ were assessed by quantification of their stable metabolites (6‐keto‐prostaglandin F1_*α*_, 6‐k‐PGF1_*α*_; thromboxane B_2_, TXB_2_), using ELISA. Dense granule ATP secretion from activated platelets was determined by bioluminescence; RH and CIMT were determined by fingertip tonometry and ultrasound, respectively. After 48 months of treatment, platelet content of 6‐k‐PGF1_*α*_ and TXB_2_ was significantly lower in oCEE compared to the PL. Inhibition of ATP secretion by exogenous activation of cAMP associated with platelet 6‐k‐PGF1_*α*_ (*r* = −0.41, *P* = 0.04) and TXB_2_ (*r* = 0.71, *P* = 0.0005) only in the oCEE group. Serum and platelet content of 6‐k‐PGF1_*α*_ and TXB_2_ associated positively in the PL and tE2 groups. Serum 6‐k‐PGF1_*α*_ positively associated with RH in the oCEE group (*r* = 0.73, *P* = 0.02), while serum TXB_2_ positively associated with CIMT in the tE2 group (*r* = 0.64, *P* = 0.01). Thus, oCEE and tE2 differentially affect prostanoid‐mediated platelet secretory pathways but alone would not account for an increased thrombotic risk for oral MHT. Furthermore, platelet‐derived prostanoids may contribute to RH and vascular remodeling in healthy menopausal women.

## Abstract (precis)

Oral conjugated equine estrogen and transdermal 17β‐estradiol differentially affect prostanoid‐mediated platelet secretory pathways. Platelet‐derived prostanoids may contribute to endothelial function and vascular remodeling in healthy menopausal women.

## Introduction

Estrogenic treatments used to reduce menopausal symptoms carry a label warning of risk for thrombosis with their use. Further, population‐based and multicenter case–control studies provide evidence that oral estrogenic products (either esterified estrogen or conjugated equine estrogen) may carry a small but greater risk (<4%) for thrombotic events such as ischemic stroke, myocardial infarction and venous thromboembolism than transdermal products (Scarabin et al. 1997, 2003; Writing, WGftWsHII 2002; Canonico et al. 2007; Shufelt et al. 2014). This difference between oral and transdermal products reflects, in part, the direct absorption of the estrogens into the enterohepatic circulation (De Lignieres et al. 1986), and thus, a greater effect on production in the liver of proteins associated with coagulation, fibrinolysis, and inflammation than transdermal products. However, estrogens also affect production of proteins through genomic actions on gene transcription in all nucleated cells including megakaryocytes, the precursors of platelets, and vascular endothelial cells (Elam et al. 1980; Bar et al. 1993; Mikkola et al. 2000; Duckles and Miller 2010). Indeed, estrogen dose‐dependently modulates cyclooxygenase activity and synthesis of prostacyclin (PGI_2_) and thromboxane A_2_ (TXA_2_) from platelets and endothelial cells (Roncaglioni et al. 1979; Chang et al. 1981; Corvazier et al. 1984; Schmitz et al. 1985; Holmsen 1986; Macdonald et al. 1988; Kahn et al. 1993; Redmond et al. 1994; Bolego et al. 1997; Mikkola et al. 2000; Canonico et al. 2007; Capone et al. 2007). Because the turnover of platelets in humans is about 10–12 day, platelets produced under conditions of either hormone deplete or replete conditions might be expected to differ in their secretory activity and secretome.

PGI_2_ and TXA_2_ exert opposing actions on platelet aggregation and vascular reactivity through mechanisms involving cyclic‐AMP (Vericel et al. 1988; Ibe et al. 1989; Dogne et al. 2000). In platelets, PGI_2_ activates adenylate cyclase leading to increased synthesis of cAMP which reduces platelet cytosolic calcium concentration and inhibits platelet activation; TXA_2_ does the opposite (Dogne et al. 2005). In vascular smooth muscle cells, similar mechanisms regulate smooth muscle contraction, proliferation, and migration (Schror 1993). Thus, their balance is critical to maintain vascular homeostasis.

Previous studies using sexually mature female pigs demonstrated that platelet dense granular ATP secretion increased following oophorectomy but was reduced by oral estrogenic treatments (either conjugated equine estrogen, 17 *β* estradiol or raloxifene) (Jayachandran and Miller 2002; Jayachandran et al. 2003, 2005a, 2005b). In addition, treatment with oral 17*β*‐estradiol increased the ratio of thromboxane to prostacyclin secreted from collagen‐activated platelets which could promote platelet aggregation and vasoconstriction (Lewis et al. 2006). These results suggest that oral estrogen could increase TXA_2_, decrease PGI_2_, or both. Similar observations have not been made in platelets from recently menopausal women using MHT. Therefore, the goal of this study was to evaluate and compare how oral conjugated equine estrogen and transdermal 17 *β*‐estradiol affect the content of PGI_2_ and TXA_2_ and secretion of ATP from dense granules in platelets derived from healthy, recently menopausal women. In addition, a second goal was to assess if platelet or serum PGI_2_ and TXA_2_ associated with endothelial mediated vascular reactivity and vascular remodeling. On the basis of results of the pig experiments outlined above, we hypothesized that: (1) serum levels of prostanoids would reflect their content in platelets, (2) oral estrogenic products would inhibit dense granule ATP secretion, and (3) serum TXA_2_ would correlate with lower endothelial function, measured by forearm reactive hyperemia, and increased vascular remodeling, as measured by carotid intima medial thickness (CIMT).

## Methods

### Study design

Participants were a subset of recently menopausal women enrolled in the Kronos Early Estrogen Prevention Study (KEEPS) at Mayo Clinic. KEEPS was a randomized, double‐blind, placebo‐controlled clinical trial to test whether MHT initiated within 3 years of menopause slowed progression of atherosclerosis, defined by changes in CIMT. Detailed inclusion and exclusion criteria for the KEEPS have been published (Harman et al. 2005). Briefly, exclusion criteria were: coronary arterial calcification score of >50 Agatston Units, smoking over 10 cigarettes per day, BMI >35 kg/m^2^, history of cardiovascular disease, low‐density lipoprotein cholesterol >190 mg/dL, triglycerides >400 mg/dL, blood glucose >126 mg/dL, uncontrolled hypertension (systolic blood pressure >150 mmHg and/or diastolic blood pressure >95 mmHg), current or recent (6 months) use of cholesterol‐lowering medications (statins, fibrate, or >500 mg/day niacin), follicle‐stimulating hormone level ≤35 ng/mL and estrogen levels ≥40 pg/mL.

Participants were randomized to one of three treatments: oral conjugated equine estrogen (oCEE, 0.45 mg/day) plus a placebo transdermal patch, transdermal 17*β*‐estradiol (tE2, 50 *μ*g/day) plus a placebo pill, or placebo (PL) pills and patch, for 48 months. Women in the active treatment groups also received oral micronized progesterone (200 mg) for the first 12 days of each month. Participants were asked to refrain from use of aspirin for 2 weeks prior to collection of blood and platelets.

### Serum analytes

After an overnight fast, venous blood was collected into glass tubes without anticoagulants, and centrifuged at 3000 g for 15 min. Aliquots of serum were stored at −80°C until analysis. Estrone (E1), estradiol (E2), and total testosterone were quantified using high‐sensitivity liquid chromatography–tandem mass spectrometry (HLC‐MS/MS); sex hormone‐binding globulin (SHBG) was quantified by two‐site chemiluminescent immunoassay, using the Immulite 2000 SHBG test. Serum PGI_2_ and TXA_2_ were examined by quantifying their stable metabolites 6‐keto‐prostaglandin F1_*α*_ (6‐k‐PGF1_*α*_) and thromboxane B_2_ (TXB_2_), respectively, using ELISA (#KA0294 Abnova, Oxford Biomedical Research, Oxford, MI).

### Platelet dense granule ATP secretion

Platelet‐rich plasma was prepared from blood collected with trisodium citrate (3.2%) and diluted to 250–500 platelets/*μ*L. Platelet dense granule ATP secretion in response to thrombin receptor agonist peptide (TRAP, 10 *μ*mol/L) was measured in the absence or presence of prostaglandin E_1_ (PGE_1_; ~500 nmol/L incubation for 10 min at 32°C) (Smith and Owen 1999; Jayachandran et al. 2011) by bioluminescence using premixed luciferin/luciferase. PGE_1_ increases platelet cAMP and thereby inhibits dense granule secretion in response to agonists, including TRAP. The degree of inhibition in the presence of PGE_1_ is a surrogate measure of endogenous activation of cAMP such that the greater the inhibition, the lower the endogenous cAMP activity. Dense body ATP secretion was expressed as attomoles (amoles)/platelet. PGE_1_ sensitivity was expressed as %inhibition of dense body ATP secretion.

### Platelet lysates

Platelet‐rich plasma (PRP) was separated from blood anticoagulated with hirudin and soybean trypsin inhibitor or sodium citrate and platelet number was quantified by Coulter counter (Jayachandran et al. 2011). Platelet lysate was prepared from washed platelets (Jayachandran and Miller 2002). Platelets were placed in 200 *μ*L lysis buffer [(#0103004‐L; RayBioTech, Norcross, GA); with 5 mmol/L TRIS, 0.5% TRITON X100, and a mixture protease inhibitors (serine, cysteine, aspartic proteases and aminopeptidases) (#P8340, Sigma, St. Louis, MO), pH 7.4], passed through a 26‐guage needle 8–10 times, then sonicated. Insoluble membrane debris was pelleted by centrifugation at 12,000 *g* for 5 min. Protein concentration in the lysate (supernatant) was measured (BCA kit #23225, Pierce Biotech, Rockford, IL), then TXA_2_ and PGI_2_ were examined by quantifying their stable metabolites TXB_2_ and 6‐k‐PGF1_*α*_, respectively, using ELISA kits (GenWay Biotech, San Diego, CA and Abnova Oxford Biomedical Research, Oxford, MI, respectively).

### Carotid intima medial thickness

Carotid intima medial thickness, the primary outcome of KEEPS, was measured by B‐mode ultrasonography (Hodis et al. 2001; Miller et al. 2009).

### Endothelial function

Endothelial function was measured using peripheral tonometry to detect changes in digital pulse volume during reactive hyperemia (EndoPAT, model 2000; Itamar Medical, Ltd., Caesarea, Israel) (Mulvagh et al. 2010). The Reactive Hyperemia Index (RHI) was calculated by dedicated software in the system computer.

### Statistical analysis

All data were collected at exit from the study, that is, after 48 months of treatment. Data are presented as mean ± SEM. The number of participants is designated by “*n*”. Differences in the number of samples analyzed in each category reflect insufficient amount of platelet lysate available to perform all of the assays. The ratio of PGI_2_ to TXA_2_ could be determined only on samples that had both measurements from the same individual. Platelet lysate concentrations of 6‐k‐PGF1_*α*_ and TXB_2_ approximated normal distribution. Not all participants elected to undergo testing for endothelial reactive hyperemia as this was an ancillary study to the KEEPS protocol and the number of data points reflects only those participants for whom there were measures of PGI_2_, TXA_2_, and reactive hyperemia.

A series of linear regression models and Pearson's correlations were used to test associations. Regression coefficients and corresponding *P*‐values were calculated for each model. One‐way analysis of variance (ANOVA) was performed for pairwise multiple comparisons of the three treatment groups, followed by Dunnett's or Holm–Sidak post hoc analysis tests; significance was accepted at *P* < 0.05. Student's *t*‐test was also used to identify differences between two treatment groups.

## Results

### General characteristics

Clinical characteristics of this subset of KEEPS participants from whom platelets were studied after 48 months of treatment are shown in Table 1.

**Table 1.** Characteristics of KEEPS participants after 48 months of treatment

	Placebo *N* = 27	tE2 *N* = 22	oCEE *N* = 20
Demographics			
Age (years)	57.1 ± 0.4	57.4 ± 0.6	56.8 ± 0.5
Months past menopause	64.5 ± 1.7	69.8 ± 1.8	65.7 ± 2.4
Cardiovascular risk factors			
Waist circumference (cm)	92.6 ± 2.0	84.5 ± 2.7	88.4 ± 3.0
Low‐density lipoprotein (mg/dL)	114.0 ± 5.5	108.8 ± 7.4	99.9 ± 7.4*
High‐density lipoprotein (mg/dL)	73.3 ± 2.5	73.6 ± 2.6	75.0 ± 2.9
Triglycerides (mg/dL)	90.1 ± 10.4	78.1 ± 7.2	120.3 ± 8.3*
Systolic blood pressure (mmHg)	120.3 ± 2.7	119.9 ± 3.7	120.9 ± 2.7
Fasting glucose (mg/dL)	81.7 ± 1.2	79.6 ± 1.6	81.9 ± 2.5
Other			
C‐Reactive protein (mg/L)	1.8 ± 0.5	1.4 ± 0.3	2.7 ± 0.6
Platelet count (10^3^/*μ*L)	237.0 ± 8.5	228.1 ± 14.1	232.9 ± 11.8
Vascular imaging			
Carotid intima medial thickness (mm)	0.70 ± 0.02	0.75 ± 0.02	0.68 ± 0.02
Reactive hyperemia index	2.50 ± 0.12	2.28 ± 0.16	2.39 ± 0.18

Data are shown as mean ± SEM.

* Denotes statistical significance from other groups, *P* < 0.05.

MHT for 48 months increased serum estrone, 17*β*‐estradiol, and sex hormone‐binding globulin compared to PL. Serum estrone was higher with oCEE than with tE2, whereas serum 17*β*‐estradiol was higher with tE2 than with oCEE. Sex hormone‐binding globulin was significantly greater in the oCEE group compared to tE2 and PL, while total testosterone was similar among groups (Table 2).

**Table 2.** Hormone levels after 48 months of treatment

Hormone	Placebo *N* = 27	tE2 *N* = 22	oCEE *N* = 20
Estrone (pg/mL)	22.6 ± 2.0	35.1 ± 2.8	66.1 ± 8.5*
17*β*‐estradiol (pg/mL)	6.4 ± 0.5	30.6 ± 6.0*	13.0 ± 1.6
Sex hormone‐binding globulin (nmol/L)	50.3 ± 5.3	58.4 ± 5.4	107.1 ± 11.4*
Total testosterone (ng/dL)	23.1 ± 3.1	25.4 ± 2.5	26.8 ± 2.8

Data are shown as mean ± SEM.

* Denotes statistical significance from other groups, *P* < 0.05.

### Effect of MHT on prostanoids in platelets and serum

**Figure 1.**
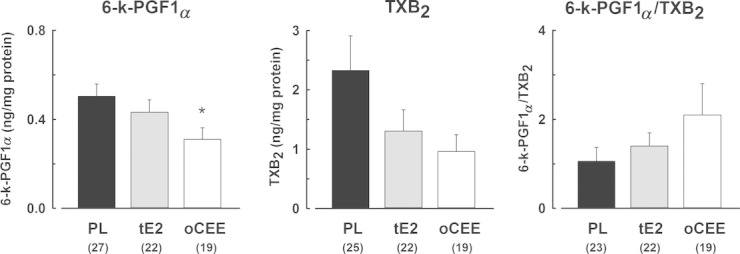
Content of stable metabolites of prostacyclin (6‐k‐PGF1_*α*_, left panel), and thromboxane (TXB_2_, middle panel), and their ratio (right panel) in platelets of menopausal women treated either with placebo (PL), transdermal 17*β*‐estradiol (tE2, 50 *μ*g/day), or oral conjugated equine estrogen (oCEE, 0.45 mg/day) for 48 months. Data are shown as means ± SEM, *n* = unique individuals from whom platelet contents were measured. Asterisk denotes significant difference from PL group, *P* < 0.05.

Platelet count did not differ significantly among groups (Table 1). Platelet content of 6‐k‐PGF1_*α*_ was significantly lower in platelets of women in the oCEE group compared to PL (Fig. 1). TXB_2_ was variable ranging 30–10950 pg/mg protein (median 1205.6 pg/mg protein) in the PL group, 54–5929 pg/mg protein (median 278.7 pg/mg protein) in the tE2 group, and 12–3944 pg/mg protein (median 305.0 pg/mg protein) in the oCEE group. Although the mean for TXB_2_ in the oCEE group was lower than the other groups (961 pg/mg protein in the oCEE group, 1303 pg/mg protein in the tE2 group and 2327 pg/mg protein in the PL group) statistical significance was not realized. The ratio of 6‐k‐PGF1_*α*_ to TXB_2_ was variable also; however, the mean ratio of 6‐k‐PGF1_*α*_ to TXB_2_ was about twice as great in the oCEE compared to tE2 or PL groups (ratio = 1.03, 0.48, 0.38, respectively).

**Figure 2.**
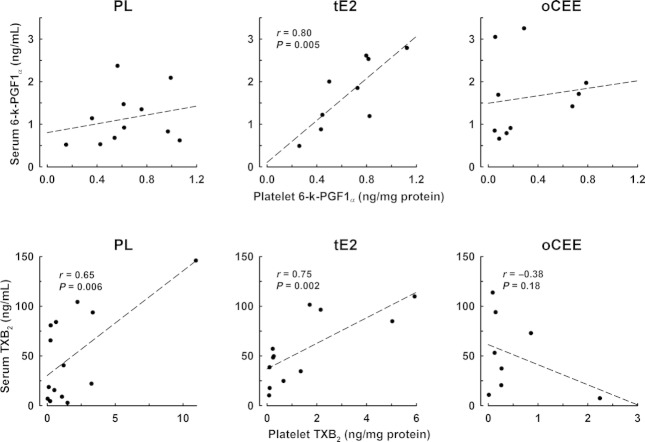
Relationships between serum and platelet levels of prostacyclin (6‐k‐PGF1_*α*_) and thromboxane (TXB_2_) in menopausal women treated with either placebo (PL), transdermal 17*β*‐estradiol (tE2, 50 *μ*g/day), or oral conjugated equine estrogen (oCEE, 0.45 mg/day) for 48 months. Each point represents an individual.

Serum levels of 6‐k‐PGF1_*α*_ and TXB_2_ did not differ among groups (data not shown). There was a positive correlation between serum and platelet levels of 6‐k‐PGF1_*α*_ only in the tE2 group (*r* = 0.80, *P* = 0.005). Serum levels of TXB_2_ correlated with platelet content in all groups (Fig. 2). In the PL and tE2 groups, this correlation was positive but in the oCEE group, there was a negative correlation suggesting less‐activated release of platelet content of TXB_2_ during spontaneous aggregation in the oCEE group.

### Effects of MHT on platelet dense granule ATP secretion

**Figure 3.**
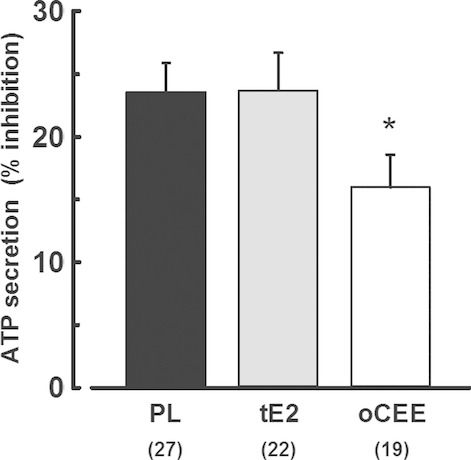
Percent inhibition of dense granule adenosine triphosphate (ATP) secretion from platelets by PGE_1_. Adenosine triphosphate secretion in response to thrombin receptor agonist peptide (TRAP 10 *μ*mol/L) was measured in tandem in paired samples of platelets from each participant in the absence and presence of PGE_1_ (~500 nmol/L). Data are shown as the percent change in ATP secretion in the presence of PGE_1_. The greater the inhibition suggests greater increase in cAMP by PGE_1_. Asterisk denotes significant difference from PL group by Student's *t*‐test, *P* < 0.05.

**Figure 4.**
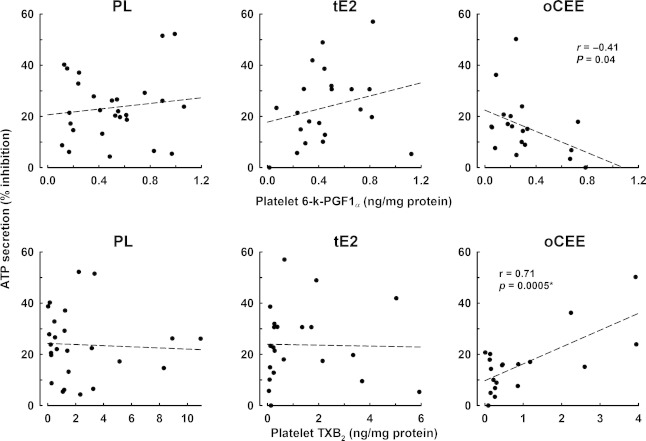
Associations of inhibition of adenosine triphosphate (ATP) secretion with platelet content of stable metabolites of prostacyclin (6‐k‐PGF1_*α*_) and thromboxane (TXB_2_) in platelets of menopausal women treated with either placebo (PL), transdermal 17*β*‐estradiol (tE2, 50 *μ*g/day), or oral conjugated equine estrogen (oCEE, 0.45 mg/day) for 48 months. Each point represents an individual.

There were no significant differences in ATP secretion in response to TRAP among treatment groups. However, ATP secretion was inhibited by PGE_1_ to a greater extent in platelets from women in the PL and tE2 groups compared to oCEE group (Fig. 3). The percent inhibition significantly correlated with platelet content of 6‐k‐PGF1_*α*_ and TBX_2_ only in platelets of women treated with oCEE (Fig. 4).

### Relationship between platelet or serum prostanoids and vascular parameters

**Figure 5.**
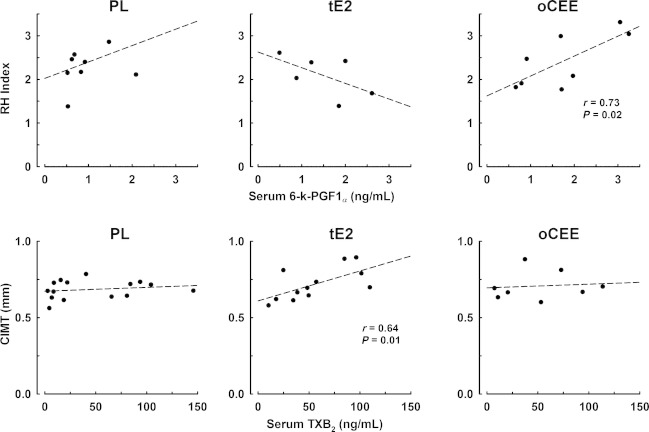
Associations of serum prostanoids with RH index (upper panels) and CIMT (lower panels) in menopausal women treated with either placebo (PL), transdermal 17*β*‐estradiol (tE2, 50 *μ*g/day), or oral conjugated equine estrogen (oCEE, 0.45 mg/day) for 48 months. Each point represents an individual.

Neither platelet content of 6‐k‐PGF1_*α*_ nor TXB_2_ correlated with RHI or CIMT in any group (data not shown). However, serum levels of 6‐k‐PGF1_*α*_ correlated with RH index only in the oCEE group (*r* = 0.73, *P* = 0.02), while TXB_2_ concentrations in serum significantly correlated (*r* = 0.64, *P* = 0.01) with CIMT only in the tE2 group (Fig. 5).

## Discussion

This study identified several differences in platelet prostanoid content and dense granule secretion in platelets derived from women using oral conjugated equine estrogen and transdermal 17*β* estradiol compared to placebo. These differences in platelet characteristics and serum levels of prostanoids affect vascular reactivity and remodeling.

### MHT platelet prostanoids and dense granule ATP secretion

Given that the risk of thrombosis is higher with the use of oral compared to transdermal estrogen products, an unexpected finding of the present study was that platelet content of TXA_2_ a procoagulant prostanoid (Scarabin et al. 1997, 2003; Canonico et al. 2007) trended to be lower in platelets from the oCEE group than the tE2. This difference could reflect either or both increased secretion or decreased production of the prostanoid. Because neither cyclooxygenase activity nor thromboxase synthase activity was measured directly in these platelets, it is not possible to draw a conclusion related to synthesis. However, the negative correlation between serum and platelet TXA_2_ in the oCEE group argues against the concept that low platelet content resulted from increased secretion during spontaneous aggregation of the platelets in vivo in this group. However, the positive correlation between serum and platelet levels of TXA_2_ in the PL and tE2 groups suggest that serum levels may reflect release of TXA_2_ from platelets during spontaneous aggregation or higher platelet content in these groups.

**Figure 6.**
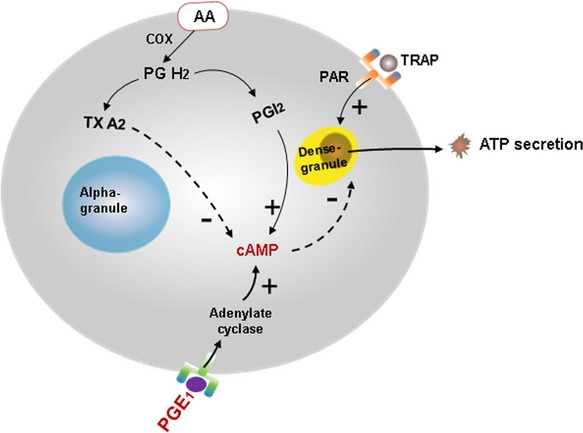
Schematic depicting regulation of dense granule ATP secretion by cytosolic prostanoids in platelets. AA, arachidonic acid; ATP, adenosine triphosphate; cAMP, cyclic adenosine monophosphate; COX, cyclooxygenase; PAR, protease‐activated receptor; PGE_1_, prostaglandin E_1_; PG H2, prostaglandin H2; PGI_2_, prostacyclin; TRAP, thrombin receptor agonist peptide; TXA_2_, thromboxane; minuses indicate inhibition, pluses indicate activation.

Platelet cytosolic levels of PGI_2_ and TXA_2_ affect platelet dense granule ATP secretion through regulation of cAMP such that increases in cAMP decrease platelet calcium concentration, rendering the platelet relatively less responsive to agonist‐stimulated granular secretion (Fig. 6). In the oCEE group, inhibition of ATP secretion by PGE_1_ was significantly lower than in the other groups. This observation is consistent with the trend to decreased content of TXA_2_ in platelet from the oCEE group. The percent inhibition of ATP secretion reflected endogenous activation of cAMP such that inhibition decreased in proportion to cytosol PGI_2_, reflecting high endogenous cAMP activity but increased in proportion to cytosolic TXA_2_, reflecting low endogenous cAMP activity. ATP secretion did not differ among groups in the absence of PGE_1_ which may reflect the maximal concentration of TRAP used to stimulate the platelets. Titered release of ATP may have been observed by using lower, threshold, concentrations of the agonist.

### Relationship among platelet and serum prostanoids and vascular parameters

Both prostacyclin and thromboxane affect vasomotion and vascular smooth muscle cell proliferation, albeit in opposite ways (Schror 1993). As mentioned above, in the present study as is consistent with other studies, serum levels of thromboxane reflected the platelet levels, but only in the PL and tE2 groups. Serum prostacyclin is thought to reflect that derived from endothelial cells rather than platelets (Callahan et al. 1986). However, in the present study, there was a positive correlation between serum and platelet prostacyclin in the tE2 group suggesting that under some conditions, platelets contribute to circulating levels of prostacyclin.

Release of thromboxane from platelets would be expected to cause vasoconstriction and reduce the RH index. However, RH index did not correlate with serum thromboxane in any group and unexpectedly, RH index correlated to serum prostacyclin only in the oCEE group. RH index will reflect the cumulative actions of other factors such as ATP and 5‐hydroxytryptamine released from activated platelets as well as factors released from the endothelium in response to increased shear stress.

Both prostanoids affect smooth muscle proliferation. In the present study, serum levels of prostanoids reflected platelet content only in the tE2 and PL groups. The relationship between serum thromboxane and CIMT in the tE2 group is consistent with the concept that platelets are a source of mitogenic factors affecting vascular remodeling. However, thromboxane and prostacyclin are only two of multiple factors released from platelets which might affect vascular remodeling at sites of endothelial dysfunction. These prostanoids may act synergistically or antagonistically with other growth factors, proteins, or amines associated with immunity, matrix deposition, proteases, and transport (Wijten et al. 2013). Therefore, the change in CIMT over time would likely reflect the collective effect of platelet and blood‐borne cytokines as well as mechanical forces on the vascular wall (Jiang et al. 2000).

### Limitations

There are several limitations to this study. First, although both oral conjugated equine estrogen and esterified estrogen may infer a greater risk for thrombosis than transdermal products (Scarabin et al. 1997, 2003; Canonico et al. 2007), the oral and transdermal products used in the present study were not the same formulation. Therefore, it is not possible to conclude definitively if the route of delivery affected the differences in response compared to the formulation. However, as circulating levels of 17*β*‐estradiol and estrone differed between the treatments groups, the effects observed in the present study most likely reflect effects of the formulation on the platelet characteristics. 17*β*‐estradiol has greater binding affinity for estrogen receptors than does estrone (Bhavnani and Woolever 1991). However, estrone can be metabolized to 17*β*‐estradiol by estradiol 17‐*β*‐dehydrogenase 1 which may provide a more prolonged concentration of estrogen and activation of estrogen receptors at the tissue level than might be reflected by serum levels of the hormone (Bagot et al. 2010). With the transdermal product used in this study, premenopausal levels of 17*β* estradiol were not achieved and further studies would be needed to evaluate a dose effect on the platelet parameters with this product. Oral formulations affect other proteins of the coagulation cascade and inflammatory proteins through actions on their synthesis in the liver (Lacut et al. 2003; Scarabin et al. 2003; Eilertsen et al. 2005; Shufelt et al. 2014). Although proteins of the coagulation cascade have yet to be measured in plasma from KEEPS participants, C‐reactive protein was elevated to a greater extent in the oCEE group. Therefore, the thrombotic risk associated with oral MHT products may not be related to their direct action on platelet responsiveness alone but related to inflammatory risk factors induced by smoking or infection that affect other components of coagulation cascade of an individual.

A second limitation is that the volume of platelet lysate obtained from each participant was variable and in some cases was insufficient to allow analysis of both prostanoids. In addition, some participants declined measurement of endothelial reactive hyperemia which was an ancillary study to the KEEPS and lowered the number of data points for this parameter.

Finally, baseline levels of serum and platelet prostanoids were not measured. Thus, the study could evaluate only cross‐sectional group effects of treatment rather than paired analysis of treatment effect over time.

## Conclusion

This study is the first to compare prostanoid levels in platelets from recently menopausal women using two different formulations of MHT in relationship with platelet secretory function and vascular parameters. Specifically, oCEE had an inhibitory effect on platelet prostanoids, which may affect platelet sensitivity to agonist activation. Serum levels of prostacyclin and thromboxane may not provide an accurate reflection of their concentration in and release from platelets. These effects of MHT on platelet secretion alone may not account for increased thrombotic risk with their use. However, serum prostanoids may affect vascular reactivity and remodeling. Differences in platelet functions with oCEE compared to tE2 may reflect variation in tissue concentrations of 17*β*‐estradiol compared to its metabolites. These metabolites through differential binding affinity for estrogen receptors will modulate genomic actions at the level of the megakaryocytes as well as nongenomic regulation of cytosolic pathways in circulating platelets.

## Acknowledgments

The authors thank the dedicated volunteers participating in this study and coworkers who made this possible including Rebecca Beck, RN, Teresa G. Zais, Robert Litwiller and the staff in the Women's Health Clinic.

## Conflict of Interest

None declared.
